# Incidence and Predictors of End Stage Renal Disease among Low-Income Blacks and Whites

**DOI:** 10.1371/journal.pone.0048407

**Published:** 2012-10-24

**Authors:** Loren Lipworth, Michael T. Mumma, Kerri L. Cavanaugh, Todd L. Edwards, T. Alp Ikizler, Robert E.Tarone, Joseph K. McLaughlin, William J. Blot

**Affiliations:** 1 Division of Epidemiology, Department of Medicine, Vanderbilt University Medical Center and Vanderbilt-Ingram Cancer Center, Nashville, Tennessee, United States of America; 2 International Epidemiology Institute, Rockville, Maryland, United States of America; 3 Division of Nephrology, Department of Medicine, Vanderbilt University Medical Center, Nashville, Tennessee, United States of America; National Cancer Institute, United States of America

## Abstract

We evaluated whether black race is associated with higher incidence of End Stage Renal Disease (ESRD) among a cohort of blacks and whites of similar, generally low socioeconomic status, and whether risk factor patterns differ among blacks and whites and explain the poorly understood racial disparity in ESRD. Incident diagnoses of ESRD among 79,943 black and white participants in the Southern Community Cohort Study (SCCS) were ascertained by linkage with the United States Renal Data System (USRDS) from 2002 through 2009. Person-years of follow up were calculated from date of entry into the SCCS until date of ESRD diagnosis, date of death, or September 1, 2009, whichever occurred first. Cox proportional hazards models were used to estimate hazard ratios (HRs) and 95% confidence intervals (CI) for incident ESRD among black and white participants in relation to baseline characteristics. After 329,003 person-years of follow-up, 687 incident cases of ESRD were identified in the cohort. The age-adjusted ESRD incidence rate was 273 (per 100,000) among blacks, 3.5-fold higher than the rate of 78 among whites. Risk factors for ESRD included male sex (HR = 1.6; 95% CI 1.4–1.9), low income (HR = 1.5; 95% CI 1.2–1.8 for income below vs. above $15,000), smoking (HR = 1.2; 95% CI 1.02–1.4) and histories of diabetes (HRs increasing to 9.4 (95% CI 7.4–11.9) among those with ≥20 years diabetes duration) and hypertension (HR = 2.9; 95% CI 2.3–3.7). Patterns and magnitudes of association were virtually identical among blacks and whites. After adjustment for these risk factors, blacks continued to have a higher risk for ESRD (HR = 2.4; 95% CI = 1.9–3.0) relative to whites. The black-white disparity in risk of ESRD was attenuated but not eliminated after control for known risk factors in a closely socioeconomically matched cohort. Further research characterizing biomedical factors, including CKD progression, in ESRD occurrence in these two racial groups is needed.

## Introduction

In the last two decades, there has been a dramatic increase in the age-adjusted incidence of end stage renal disease (ESRD) in the United States, from 219 per million in 1991 to 355 per million in 2009 [1]. Since 2000, the rate of new ESRD cases has increased 7.2% among whites, while remaining stable among blacks [1], but in 2008 incidence for blacks was 3.5 times greater than for whites [1]–[3]. ESRD patients have a striking impact on national Medicare costs, which covers health care for incident ESRD in the United States regardless of age. At present, Medicare is the primary payer for 83% of hemodialysis patients, and total Medicare spending for treatment of ESRD in the United States is $29 billion, over 6% of the Medicare budget [1], and is expected to continue its rapid rise, in part due to the continued increasing prevalence of ESRD.

Although the higher rates of ESRD among blacks compared with whites are well-known, reasons for the increased ESRD risk among blacks and the extent to which differences in socioeconomic factors and other known determinants of ESRD may account for the observed racial difference in incidence is more limited. A recent study conducted within the San Francisco Community Health Network, which serves an urban poor population, reported that among adults with non-dialysis dependent chronic kidney disease (CKD), 73% of whom had an annual income below $15,000, blacks had a substantially higher risk for progression to ESRD than whites after adjustment for socioeconomic variables and medical risk factors [4]. The Southern Community Cohort Study (SCCS) is a large, prospective cohort study examining health disparities among adults, over two-thirds black, residing in the southeastern United States where rates of ESRD are among the highest in the nation [1]. The SCCS provides unique advantages for examining differences in ESRD, since both black and white participants have similar (typically low) income and education levels, so that socioeconomic differences that sometimes confound racial comparisons are minimized. Thus, we have characterized and compared the incidence of ESRD among black and white participants in the SCCS, taking into account extensive well-defined data on socioeconomic factors, and we have examined race-specific associations between baseline demographic, lifestyle and medical factors and ESRD to enhance understanding of the independent determinants of ESRD among blacks versus whites.

## Methods

### Ethics statement

SCCS participants provided written informed consent, and protocols were approved by the Institutional Review Boards of Vanderbilt University Medical Center and Meharry Medical College.

### Study population

The SCCS is an ongoing, prospective cohort study which enrolled nearly 86,000 adults, age 40–79, residing in 12 states in the southeastern United States during 2002–2009. Approximately 86% of participants were enrolled at participating community health centers (CHCs), institutions which provide primary health and preventive services in medically underserved areas and thus serve generally low-income populations [5], and the remaining 14% through mail-based general population sampling. The SCCS study design and methods have been described in detail previously [6].

Upon entry into the SCCS, participants were administered a baseline computer-assisted personal interview at the CHC while general population participants completed a self-administered mailed questionnaire. The questionnaire (available at www.southerncommunitystudy.org) ascertained information about demographic and socioeconomic characteristics, personal and family medical history, height, weight, tobacco and alcohol use history, and other factors. Many of the questions on the SCCS questionnaire were adapted from questionnaires used and validated in other settings, and a series of independent validation studies using biomarkers, repeat interviews or medical records have demonstrated the reliability of the questionnaire within the SCCS population for variables such as tobacco use status, self-reported diseases including diabetes, height and weight [6].

### Outcome ascertainment

Incident diagnoses of ESRD among SCCS participants after entry into the cohort were ascertained by linkage, using Social Security number, date of birth, and first and last name, with the United States Renal Data System (USRDS) from January 1, 2002 through September 1, 2009 (the latest date for which data were available), providing virtually complete ascertainment of all persons in the United States receiving treatment for ESRD. Person-years of follow up for ascertainment of ESRD were calculated from date of entry into the SCCS until the date of diagnosis of ESRD, date of death, or September 1, 2009, whichever occurred first. Mortality (and date and cause of death) was ascertained by linkages with the Social Security Administration vital status service for epidemiologic researchers and the National Death Index through December 31, 2009. A total of 404 individuals with a diagnosis of ESRD recorded in the USRDS prior to enrollment in the SCCS (prevalent cases) were excluded from this analysis. Twenty-nine SCCS participants who did not have an incident diagnosis of ESRD identified through USRDS but had ESRD (ICD-10 N180, I120, I131, I132) listed as an underlying or contributing cause of death were not considered incident ESRD diagnoses, as misclassification of acute kidney injury could not be ruled out, and these 29 participants were excluded from the analysis.

### Statistical analyses

Analyses were restricted to self-reported African American or black and non-Hispanic white SCCS participants who enrolled prior to September 1, 2009, since too few persons in other racial groups were available for stable statistical analysis. Percentage distributions of ESRD cases and age-adjusted (US 2000 standard population) incidence rates of ESRD were calculated in relation to demographic, socioeconomic, lifestyle, anthropometric and medical history characteristics reported at baseline, overall and by race and sex. Cox proportional hazards models, using age as the time scale, were used to estimate hazard ratios (HRs) and corresponding 95% confidence intervals (CI) for incident ESRD separately among black and white participants, and for both races combined, in relation to the following baseline characteristics: sex (male, female); recruitment source (CHC/general population); education (<12^th^ grade, high/vocational school, some college or more); annual household income (<$15,000, ≥$15,000); cigarette smoking status (ever, never); history of diabetes (no/duration <10 years/duration 10–19 years/duration ≥20 years), hypertension, stroke, high cholesterol, and myocardial infarction (MI)/coronary artery bypass (CABG) (all yes/no). Tests for interaction of these variables with race were conducted by adding the corresponding cross-product terms to the models, and significance was based on two-sided tests with a nominal significance level of 0.05. Statistical analyses were conducted using SAS software, version 9.2 (SAS Institute Inc., Cary, NC).

## Results

Among the 79,943 SCCS participants included in this analysis, 68% (N = 54,751) were black and 32% (N = 25,192) were white, and approximately 60% were women. Mean ages at the beginning of follow-up were 51.3 and 53.9 years among blacks and whites, respectively. Overall, 55% of participants had a household income below $15,000, and 29% reported an education level below high school. A diagnosis of diabetes, hypertension or MI/CABG was reported by 21%, 56% and 7% of participants, respectively.

After 329,003 person-years of follow-up, 687 incident cases of ESRD were identified in the cohort, yielding an overall age-adjusted incidence rate (IR) of 214 (per 100,000 person-years). The distribution and age-adjusted incidence rates of ESRD in relation to baseline characteristics are reported in Table 1. Six hundred and six (88.2%) of the ESRD cases were among blacks, yielding an incidence rate of 273, higher than the incidence rate of 78 among whites. Approximately 47% of the cases occurred among men, although the incidence of ESRD was higher among men (IR = 242) than women (IR = 194). Further, the substantially higher rate of ESRD among blacks than whites was apparent in both men and women, with rates (per 100,000) of 308, 248, 90 and 70 among black men, black women, white men and white women, respectively (data not shown).

**Table 1:** Distribution and age-adjusted (US 2000 standard) incidence rates (per 100,000) of end-stage renal disease (ESRD) in relation to baseline characteristics of 79,943 black and white SCCS participants, 2002–2009 (excluding those with prevalent ESRD at baseline).

	Overall		African American		White	
Characteristic	N (%)	IR	N (%)	IR	N (%)	IR
**Total**	687 (100)	214	606 (100)	273	81 (100)	78
**Recruitment**						
CHC Population	635 (92)	230	569 (94)	279	66 (81)	89
General Population	52 (8)	126	37 (6)	216	15 (19)	59
**Age at enrollment (years)**						
40–49	258 (38)	244	240 (40)	288	18 (22)	41
50–59	249 (36)	223	214 (35)	273	35 (43)	89
60–69	138 (20)	335	113 (19)	432	25 (31)	120
70–79	42 (6)	242	39 (6)	349	3 (4)	52
**Sex**						
Female	367 (53)	194	324 (53)	247	43 (53)	69
Male	320 (47)	242	282 (47)	308	38 (47)	90
**Marital status**						
Married	213 (31)	190	177 (29)	276	36 (44)	68
Separated/Divorced	222 (32)	203	194 (32)	224	28 (35)	134
Widowed	100 (15)	251	90 (15)	314	10 (12)	69
Single Never Married	149 (22)	315	142 (24)	368	7 (9)	87
**Education**						
Less than 12th grade	268 (39)	272	244 (40)	322	24 (30)	102
High/Vocational School	268 (39)	200	239 (39)	261	29 (36)	66
Some college or more	151 (22)	139	123 (20)	176	28 (35)	72
**Annual household income ($)**						
less than $15,000/year	482 (71)	280	429 (72)	328	53 (65)	123
$15,000/year or more	195 (29)	128	167 (28)	179	28 (35)	43
**Health insurance**						
No Insurance	224 (33)	140	201 (33)	171	23 (29)	56
Any Private/CHAMPUS/Other	127 (19)	122	107 (18)	173	20 (25)	47
Medicaid/Medicare Only	332 (49)	332	295 (49)	392	37 (46)	136
**BMI at Enrollment (kg/m^2^)**						
Underweight (<18.5)	7 (1)	201	6 (1)	260	1 (1)	73
Normal (18.5–24.9)	154 (23)	224	139 (23)	315	15 (19)	72
Overweight (25–29.9)	180 (27)	174	164 (28)	233	16 (20)	44
Obese (30+)	332 (49)	237	284 (48)	284	48 (60)	106
**BMI at Age 21 (kg/m^2^)**						
Underweight (<18.5)	57 (9)	206	53 (10)	290	4 (5)	43
Normal (18.5–24.9)	312 (49)	164	277 (50)	215	35 (45)	56
Overweight (25–29.9)	143 (23)	234	126 (23)	284	17 (22)	102
Obese (30+)	120 (19)	605	98 (18)	687	22 (28)	303
**Smoking**						
Ever	452 (66)	219	396 (65)	284	56 (69)	83
Never	234 (34)	202	209 (35)	252	25 (31)	68
**Alcohol drinking**						
None	405 (60)	252	342 (58)	311	63 (80)	113
Moderate (1–3/day)	195 (29)	147	182 (31)	202	13 (16)	31
Heavy (>3/day)	70 (10)	197	67 (11)	277	3 (4)	32
**MI/CABG**						
No	580 (85)	194	521 (87)	249	59 (73)	61
Yes	103 (15)	452	81 (13)	614	22 (27)	200
**Hypertension**						
No	91 (13)	67	76 (13)	90	15 (19)	31
Yes	596 (87)	320	530 (87)	385	66 (81)	123
**Diabetes**						
No	245 (36)	103	219 (36)	136	26 (32)	29
Yes	442 (64)	611	387 (64)	716	55 (68)	283
**Hypertension and Diabetes**						
Both HTN and Diabetes	402 (59)	707	353 (58)	814	49 (60)	341
Diabetes Only	40 (6)	277	34 (6)	369	6 (7)	103
HTN Only	194 (28)	160	177 (29)	203	17 (21)	39
Neither HTN or Diabetes	51 (7)	39	42 (7)	44	9 (11)	25
**Stroke**						
No	592 (86)	199	525 (87)	255	67 (83)	68
Yes	95 (14)	447	81 (13)	556	14 (17)	228
**High cholesterol**						
No	343 (50)	162	314 (52)	205	29 (36)	41
Yes	343 (50)	306	292 (48)	404	51 (64)	128

A total of 258 cases of ESRD were observed among those aged 40–49 years at enrollment, yielding an IR of 244, which increased to an IR greater than 335 for those aged 60–69 at enrollment, but then dropped off to an IR of 242 at the oldest ages, likely reflecting a survivor effect (Table 1). The incidence of ESRD was inversely related to educational level and income. The IR of ESRD was approximately two-fold higher among those with less than 12 years of education (IR = 272) compared with those who had attended college (IR = 139), and among those with annual household income less than $15,000 (IR = 280) compared with higher income levels (IR = 128).

The association between BMI at enrollment and ESRD incidence varied by race. Black men and women who reported being overweight or obese at enrollment had similar or lower rates of ESRD compared with those with normal BMI; among whites, rates were higher among men and women who were obese at enrollment compared with those of normal BMI, but remained substantially lower than for blacks across all categories of BMI. Self-reported BMI at age 21, however, was strongly and positively associated with ESRD in both races, with a greater than three-fold overall increase from a rate of 164 among those with normal BMI up to 605 among those who were obese at age 21. The association between BMI at age 21 and ESRD was more pronounced among whites, but the ESRD rate rose as high as 687 among blacks who reported having been obese at age 21, and as high as 1139 for black women when they were considered separately (data not shown).

Finally, the incidence of ESRD was strongly associated with history of MI/CABG, hypertension, diabetes, stroke and high cholesterol, overall and in each race stratum. In particular, the incidence of ESRD among those with hypertension or diabetes was five-fold and six-fold higher, respectively, than among those without, and was eighteen times higher among those with both hypertension and diabetes compared with those who had neither. Among blacks with both hypertension and diabetes, the incidence rate of ESRD reached 814.

Table 2 presents HRs and 95% CIs for the association between ESRD and selected baseline characteristics for the study population. Men and women were combined for these analyses as there were no material differences among estimates by sex. After adjustment for age, sex, education, income, smoking, and history of hypertension, diabetes and other medical conditions, blacks continued to have a higher risk for ESRD, with a HR of 2.4 (95% CI = 1.9–3.0) relative to whites. Women were at lower risk of ESRD than men (HR = 0.6; 95% CI 0.5–0.7), and smokers had a modestly increased risk for ESRD compared with non-smokers (HR = 1.2; 95% CI 1.02–1.4). Income was inversely associated with ESRD, with a HR of 1.5 (95% CI 1.2–1.8) for those with income below $15,000, while level of education was no longer associated with ESRD in the adjusted model. For all of these variables, patterns and magnitudes of association were virtually identical among blacks and whites (Table 2). In this multivariate analysis, the inclusion of BMI, either at time of enrollment or at age 21, had no material effect on the HR estimates for the other examined covariates.

**Table 2:** Cox proportional hazard ratios (HR) and 95% confidence interval (CI) for end-stage renal disease (ESRD) in relation to baseline characteristics of black and white SCCS participants, 2002–2009 (excluding those with prevalent ESRD at baseline).

	Overall			Black			White		
Characteristic	ESRD events	HR	95% CI	ESRD events	HR	95% CI	ESRD events	HR	95% CI
**Recruitment**									
CHC Population	616	1.2	0.8–1.6	551	1.2	0.8–1.7	65	1.1	0.6–2.1
General Population	46	Ref		31	Ref		15	Ref	
**Sex**									
Female	357	0.6	0.5–0.7	314	0.6	0.5–0.7	43	0.7	0.4–1.02
Male	305	Ref		268	Ref		37	Ref	
**Race**									
Black	582	2.4	1.9–3.0						
White	80	Ref							
**Education**									
Less than 12th grade	256	0.9	0.8–1.1	232	0.9	0.8–1.1	24	0.9	0.5–1.6
High/Vocational School	259	Ref		230	Ref		29	Ref	
Some college or more	147	0.9	0.7–1.1	120	0.8	0.7–1.03	27	1.0	0.6–1.8
**Annual Household Income**									
less than $15,000/year	472	1.5	1.2–1.8	420	1.4	1.2–1.7	52	1.6	0.97–2.8
$15,000/year or more	190	Ref		162	Ref		28	Ref	
**Smoking**									
Ever	437	1.2	1.02–1.4	382	1.2	1.02–1.5	55	1.2	0.7–1.9
Never	225	Ref		200	Ref		25	Ref	
**Hypertension Dx**									
No	90	Ref		75	Ref		15	Ref	
Yes	572	2.9	2.3–3.7	507	3.0	2.3–3.9	65	2.1	1.1–3.8
**Time Since Diabetes Dx**									
Never Diagnosed	240	Ref		214	Ref		26	Ref	
less than 10 years	134	2.6	2.1–3.3	118	2.6	2.1–3.3	16	2.9	1.5–5.5
10–19 years	176	8.7	7.1–10.7	153	8.4	6.7–10.5	23	10.9	6.0–19.9
20 or more years	112	9.4	7.4–11.9	97	8.9	6.9–11.6	15	13.2	6.7–26.1
**MI/CABG**									
No	563	Ref		504	Ref		59	Ref	
Yes	99	1.3	1.00–1.6	78	1.3	0.97–1.6	21	1.2	0.7–2.1
**Stroke**									
No	569	Ref		503	Ref		66	Ref	
Yes	93	1.3	1.1–1.7	79	1.3	1.1–1.7	14	1.3	0.7–2.3
**High cholesterol**									
No	334	Ref		305	Ref		29	Ref	
Yes	328	1.2	1.01–1.4	277	1.2	1.01–1.4	51	1.2	0.7–1.9

History of diabetes was the strongest predictor of ESRD in our study population, both overall and separately by race. Compared with those who had never been diagnosed with diabetes, HRs increased from 2.6 (95% CI 2.1–3.3) to 8.7 (95% CI 7.1–10.7) to 9.4 (95% CI 7.4–11.9) among those with a duration of diabetes of less than 10, 10–19, and 20 or more years, respectively (Table 2). Hypertension was also strongly associated with ESRD, with a nearly three-fold increased risk (HR = 2.9; 95% CI 2.3–3.7) among those who reported a diagnosis of hypertension at baseline. History of MI/CABG, stroke and high cholesterol were also modestly associated with ESRD overall, with HRs of 1.3 (95% CI 1.00–1.6), 1.3 (95% CI 1.1–1.7) and 1.2 (95% CI 1.01–1.4). For no risk factor was the formal test for interaction by race statistically significant, indicating that the ESRD HRs associated with the factors were similar between blacks and whites.

## Discussion

In this population of socioeconomically similar black and white SCCS participants, the age-adjusted incidence of ESRD was more than 3.5 times higher among black than whites. Even after controlling for well-defined socioeconomic factors and extensive demographic, medical and lifestyle information collected at study entry, including comorbidities such as diabetes and hypertension, blacks continued to have a 2.4-fold greater risk of ESRD relative to whites. It is noteworthy that the strengths of the associations of the various factors with risk of ESRD tended to be the same for blacks and whites. Thus, although residual confounding cannot be ruled out entirely, the examined socioeconomic and other risk factors for ESRD do not explain the observed substantially higher incidence of ESRD observed among blacks.

Our data are consistent with national statistics [1] and observations of higher rates of ESRD observed among blacks in other study populations [7]–[9]. A small number of prospective studies have had sufficient sample size and length of follow up to directly compare risk factors for incident ESRD among blacks and whites. In the Atherosclerosis Risk in Communities (ARIC) study [7] of 3,954 blacks and 11,370 whites, older age, smoking, male sex, diabetes and hypertension were positively associated with incident ESRD, and African American race remained a strong predictor even after controlling for these factors, with a HR of 2.5. These results are very similar to what we found in the SCCS, and to results of the Multiple Risk Factor Intervention Trial (MRFIT) and a study among Medicare beneficiaries aged 66 years or older with hypertension or diabetes. [10]. Similarly, in. In a large population of veterans [8], including 311,790 blacks and 1,704,101 whites, the incidence of ESRD was consistently higher among blacks than whites at all levels of baseline kidney function, after adjustment for socioeconomic variables and comorbidities. Although that study included a larger number of ESRD cases (N = 4,379 cases among blacks, 10,769 among whites) than our study population, it did not report associations of ESRD with characteristics of the study participants other than estimated glomerular filtration rate (eGFR). Most recently, in the Reasons for Geographic and Racial Differences in Stroke (REGARDS) study, a total of 133 incident cases of ESRD were observed over a median of 3.6 years of follow-up among 27,911 participants [9]. ESRD rates among blacks and whites were comparable to those observed in the current study, but the fourfold greater risk of developing ESRD among blacks compared with whites decreased to 1.4 after adjustment for hypertension, diabetes, income, and education, as well as eGFR and albumin-to-creatinine ratio.

A striking pattern of racial differences exists also for CKD, and provides evidence that the observed disparity in ESRD incidence between blacks and whites may be due, at least in part, to racial differences in the rate of progression of CKD to ESRD. In the San Francisco Community Health Network study population, most similar to the SCCS population in terms of socioeconomic distribution, blacks with CKD stages 3 to 5 had a four-fold higher risk of progression to ESRD than whites after adjustment for socioeconomic status [4]. The prevalence of early stage CKD is higher among whites compared with blacks [11]–[14], with detailed analyses based on eGFR measurements showing that the white excess for mild CKD gives way to an excess for moderate to severe CKD in blacks [14]–[16]. It has been hypothesized that this “cross-over” pattern may be due to lower mortality at higher GFR levels among blacks. Empirical evidence, however, demonstrates that death rates are higher among blacks than whites at all levels of CKD prior to ESRD [8], [10], and rates of ESRD incidence are nearly five times higher than rates of cardiovascular death among blacks with hypertension [17], and thus does not lend support to this explanation. More intense surveillance among whites than blacks could contribute to elevated prevalence among whites of early stage CKD, for whom symptoms are less marked, or, on the contrary, whites may become symptomatic at higher GFR levels and thus have CKD diagnosed earlier. Shorter sojourn times in early stage disease by blacks than whites may also be involved, but explanations for speculated faster transitions to moderate and advanced CKD and potentially to ESRD for blacks remain clear.

We did not routinely measure baseline serum creatinine for estimation of GFR among SCCS participants, but blood samples were collected from over half of those who enrolled in CHCs. As part of ongoing nested case-control studies for breast, lung, colorectal and prostate cancer, independent of the current ESRD analysis, prediagnostic blood from 1,593 cases and age- and race-matched controls was assayed for several biomarkers, including serum creatinine. Wwe found that the mean creatinine levels were about 3% higher among black (1.10 mg/dl) than white men (1.07 mg/dl) and 14% higher among black (0.90 mg/dl) than white women (0.79 mg/dl), consistent with reports in other populations of blacks and whites [18], [19]. Using the CKD-EPI equation to estimate GFR [20], the percentages in the SCCS sample of participants with eGFR of >90 (“normal”), 90–61 and ≤60ml/min/1.73 m^2^ were 56.9%, 34.5% and 8.6% among black men, 39.5%, 51.3% and 9.2% among white men, 58.7%, 32.5% and 8.8% among black women and 47.7%, 39.5% and 12.8% among white women. These figures suggest that the overall prevalence of CKD at entry into the SCCS may be lower among blacks than whites. For severe CKD, defined by eGFR <30 ml/min/1.73 m^2^, a modest excess among blacks became apparent, with prevalences of 1.9%, 1.8%, 1.7% and 0.4% among black men, black women, white men, and white women, respectively, although based on very small numbers of study subjects. However, the observed excess in the incidence of ESRD among blacks in our study population was much greater than the excess in estimated severe CKD. Twenty-four of the incident ESRD cases in this study were among those who had existing baseline serum creatinine measurements; for these eGFR at study entry was below 90 ml/min/1.73 m^2^ for 21 (88%) and below 60 ml/min/1.73 m^2^ for 16 (67%). Further assessment of CKD is beyond the scope of this analysis, but additional research is needed using serum and urinary biomarkers collected at baseline, in conjunction with repeat blood collections to measure progression rates and times by race among SCCS participants with CKD.

While awareness and treatment of hypertension has improved among blacks over time, a substantially higher prevalence of hypertension among blacks compared with whites has been reported to persist, as well as the difference between blacks and whites in the proportion of patients with hypertension who are receiving treatment [21], [22]. In the overall SCCS population, the prevalence of self-reported hypertension at baseline was 59% among blacks, significantly higher than the 50% among whites, but approximately 80% of both blacks and whites with hypertension reported use of anti-hypertensive medications. It has been suggested that a lower blood pressure level may be necessary to slow the decline of renal function among blacks with CKD and hypertension compared with whites [23], [24], but racial differences in the susceptibility to renal damage from elevated blood pressure have been reported to persist even after adjustment for differences between blacks and whites in hypertension and hypertension-control [25]. In our study, the HR estimate associated with hypertension was somewhat (but not significantly) higher among blacks than whites (HR = 3.0 versus 2.1), which lends some support to the view that the higher incidence of ESRD in blacks may be attributable in part to a greater sensitivity to the effects of elevated blood pressure among blacks. With respect to diabetes, the onset of albuminuria, hypothesized to be an etiologic factor rather than simply a biomarker [26], appears to present earlier in the course of diabetes in blacks compared with whites, which may suggest more aggressive disease progression [27], [28], but this is unlikely to explain the similar HR estimate observed between diabetes and ESRD among whites and blacks in our study population.

The SCCS is a unique cohort in which to study health effects in blacks compared with whites both because of the large number of blacks and the comparability of socioeconomic status between the racial groups. In addition, the collection of extensive baseline information for the entire SCCS cohort and the unbiased and virtually complete follow up for ascertainment of ESRD [29] are major strengths of our study. Over 85% of our study population was drawn from CHCs, which are expected to provide race-neutral care for diabetes, hypertension and other chronic illnesses. Limitations include the lack of data on baseline kidney function or proteinuria for the ESRD cases, the self-reported nature of the questionnaire data, and the lack of time-dependent covariates. However, we conducted internally valid comparisons of HRs for ESRD between blacks and whites, and our findings support the existing literature on this subject and warrant further etiologic research in future prospective studies of ESRD in blacks.

While race appears to be an independent predictor of ESRD, and possibly of more rapid loss of kidney function among those with moderate and advanced CKD [30], longitudinal studies with repeated measures of kidney function are critically needed to evaluate whether stage-specific kidney disease may progress more rapidly in blacks than in whites and to identify genetic, environmental or behavioral factors that may explain these differences in progression rate and incidence of ESRD among blacks in the United States. A recent study [31] showed that, among those of recent African ancestry, focal segmental glomerulosclerosis and hypertension-attributed ESRD are strongly associated with two coding sequence variants in the APOL1 gene on chromosome 22. The APOL1 genotype has also been shown to associate with microalbuminuria among nondiabetics [32], and with younger age at initiation of hemodialysis among nondiabetic blacks with ESRD [33]. The APOL1 risk alleles for renal disease occur in more than 30% of those with recent African ancestry, but to date have not been observed in European Americans [31], [34]. Thus, these emerging data support the existence of a relatively common, high-risk genotype that confers susceptibility to nondiabetic kidney disease in African Americans [34].

Our study has demonstrated that, after taking into account traditional risk factors strongly associated with ESRD among both blacks and whites, blacks continue to have a substantially increased risk for ESRD compared with whites. Further research characterizing CKD progression and ESRD occurrence by race may help greatly in clarifying the natural history and etiologic events leading to ESRD in these two populations.
